# Changes in insulin‐like growth factor‐I and ‐II associated with fat but not lean mass in early old age

**DOI:** 10.1002/oby.21002

**Published:** 2015-02-03

**Authors:** David Bann, Jeff M.P. Holly, Hany Lashen, Rebecca Hardy, Judith Adams, Diana Kuh, Ken K. Ong, Yoav Ben‐Shlomo

**Affiliations:** ^1^ MRC Unit for Lifelong Health and Ageing, University College LondonLondonUK; ^2^ IGFs and Metabolic EndocrinologySchool of Clinical Sciences, Bristol UniversityBristolUK; ^3^ Department of Human MetabolismThe University of SheffieldSheffieldUK; ^4^ Department of RadiologyCentral Manchester University Hospital NHS Foundation Trust, Manchester Academic Health Science CentreOxford RoadManchesterUK; ^5^ MRC Epidemiology UnitUniversity of CambridgeCambridgeUK; ^6^ School of Social and Community MedicineBristol UniversityBristolUK

## Abstract

**Objective:**

To test the hypothesis that insulin‐like growth factors‐I and II (IGF‐I and II) decline during late midlife and that greater declines are related to higher fat mass and lower lean mass.

**Methods:**

A total of 1,542 men and women in a British birth cohort study had IGF‐I and II measured by immunoassay of blood samples at age 53 and/or 60‐64 years. Fat mass, android:gynoid fat ratio, and appendicular lean mass were measured at 60‐64 years using dual‐energy X‐ray absorptiometry (DXA). Associations between changes in IGF‐I or II and body composition outcomes were examined using conditional change linear regression models.

**Results:**

Mean IGF‐I and IGF‐II concentrations were lower at 60‐64 than at 53 years, by 12.8% for IGF‐I and by 12.5% for IGF‐II. Larger declines in either IGF‐I or II were associated with higher fat mass at 60‐64 years. Although higher IGF‐I at 53 years was associated with higher lean mass, there was little evidence linking changes in IGF‐I or II to lean mass.

**Conclusions:**

The findings suggest that IGF‐I and II concentrations decline with age, and greater declines are associated with higher fat mass levels. These results provide some evidence for the suggested roles of IGF‐I and II in regulating fat mass but not lean mass in older age.

## Introduction

Insulin‐like growth factors‐I and II (IGF‐I and II) are known to regulate growth in early life and are postulated to have multifaceted roles in adult life 1, 2. Evidence from multiple sources suggests that these roles could include the regulation of body composition (fat and lean (muscle) mass). This is important to understand given the high worldwide prevalence of obesity and its well‐documented adverse consequences for health and physical functioning 3, 4, and the likely independent adverse effects of low lean mass in old age 5.

Animal studies have suggested that IGF‐I may facilitate the maintenance of muscle satellite cells, which aid exercise‐induced muscle hypertrophy 6, 7. Small experimental studies in humans have shown that supplementation of growth hormone (the up‐stream physiological regulator of IGF‐I) leads to losses in fat and gains in lean mass 8, and Laron syndrome (genetic insensitivity to growth hormone) is characterized by high fat mass levels which are reversed by IGF‐I therapy 9. Experimental studies have also shown that obese people have blunted IGF‐I generation in response to growth hormone stimulation, suggesting bi‐directionality in the association between IGF‐I and fat mass 8.

Although IGF‐II concentrations in adults are three‐ to fivefold higher than IGF‐I concentrations 10, its roles are less well understood 11, particularly because IGF‐II is not expressed postnatally in mouse models unlike in humans. Genetic and epigenetic studies have suggested that IGF‐II may regulate fat and lean mass: IGF‐II genetic variants have been associated with body weight 12 and lean mass 13, and epigenetic differences in IGF‐II have been associated with skinfold thickness 14.

Observational studies can contribute to understanding how IGF‐I and II concentrations change with age, and how these changes impact on body composition. Limited evidence from cross‐sectional studies shows that IGF‐I concentrations are lower in old age 15, 16, 17, 18, suggesting that the decline in IGF‐I may be implicated in age‐related gains in fat and losses in lean mass 19. IGF‐I has been cross‐sectionally inversely associated with body mass index (BMI) or body weight 20, with some studies reporting that those with lowest or highest BMI have lower IGF‐I 21, 22. However, such findings do not elucidate whether IGF‐I is associated with fat and/or lean mass. Cross‐sectional studies of body composition measures have yielded equivocal findings with IGF‐I, and few have examined associations with IGF‐II 23, leading to uncertainty in the roles of IGF‐I and II in regulating body composition.

The objectives of this study were to examine the relevance of age‐related changes in circulating IGF‐I and II concentrations during late midlife to fat and lean body mass in a British birth cohort study. The strengths of this study are the repeat measures of IGF‐I and II, 7‐11 years apart, a large sample of both sexes, and measures of body composition obtained in early old age using dual‐energy X‐ray absorptiometry (DXA). We hypothesized that larger decline in IGF‐I and II would be associated with higher fat and lower lean mass.

## Methods

### Study sample

The MRC National Survey of Health and Development (NSHD) is a socially stratified sample of 5,362 singleton births that took place in 1 week of March 1946 in mainland Britain, with regular follow‐up across life. Between 2006 and 2010 (at 60‐64 years), 2,856 eligible study members (those known to be alive and with a known address in England, Scotland, or Wales) were invited for an assessment at one of six clinical research facilities (CRFs) or to be visited by a research nurse at home. Invitations were not sent to those who had died (*n* = 778), who were living abroad (*n* = 570), had previously withdrawn from the study (*n* = 594) or who had been lost to follow‐up (*n* = 564). Of those invited, 2,229 (78%) were assessed: 1,690 (59.2%) attended a CRF and the remaining 539 were seen at home 24.

### Body composition measurement

During the visits to the CRF, measures of body composition were obtained in the supine position using a QDR 4500 Discovery DXA scanner (Hologic, Bedford, MA) with APEX 3.1 analysis software. From these scans, measures of fat (whole body, abdominal (android) and hips (gynoid)) and appendicular (limb) lean mass were obtained and converted into kilograms. The ratio of android:gynoid fat mass was derived (higher values indicating greater fat distribution in the abdomen than hips) and multiplied by 100. Lean mass was defined as body mass excluding fat mass and bone mineral content (BMC), and in all measures data from the head were excluded due to the high proportion of BMC known to affect the accuracy of soft‐tissue measures. Measures selected for analysis were: whole body fat mass (kg), android:gynoid ratio (multiplied by 100), and appendicular lean mass (kg). Data on these outcomes were available for 1,558 participants, with missing data in 132 participants largely due to the presence of high‐density artefacts (e.g., joint replacements). The study received multicenter research ethics committee approval, and informed consent was given by participants.

### Measurement of IGFs

At 53 years, non‐fasting venous blood samples were taken in tubes containing ethylenediaminetetraacetic acid (EDTA) during home visits by research nurses. These were posted overnight to a laboratory where plasma was extracted and frozen at −80°C. At 60‐64 years, overnight fasting venous blood samples were obtained in tubes containing liquid citrate. These were then taken immediately to a laboratory in each CRF (clinic visit) or posted overnight to a CRF (home visit) and plasma extracted before being frozen at −80°C. IGF‐1, IGF‐II, and IGFBP‐3 were obtained by radioimmunoassay using standardized protocols in the same laboratory; in all except 200 pilot samples, assays were conducted in duplicate and mean values used in analyses. Intra‐assay coefficients of variation were 3.4% for IGF‐I, 2.8% for IGF‐II, and 3.9% for IGFBP‐3. Assays were repeated where intra‐assay coefficients of variation exceeded 15%. Inter‐assay coefficients of variation were 13.7% (IGF‐I), 7.4% (IGF‐II), and 11.7% (IGFBP‐3); to minimize inter‐assay variability, in most instances the same laboratory technician assayed each analyte.

### Analytical strategy

As taller individuals tend to have more fat and lean mass, height‐adjusted indices were created by dividing fat and appendicular lean mass (kg) by height(m)^*X*^, where *X* was calculated so that the resulting index was not correlated with height (*X* = 1.2 for fat and 2 for lean mass) 25.

First, the associations of IGF‐I and IGF‐II at 53 and 60‐64 years with fat and lean mass at 60‐64 years were examined using linear regression. To aid the interpretation of model coefficients, each IGF measure was converted into sex‐specific *z*‐scores, and outcomes were log transformed and multiplied by 100; regression coefficients therefore show the mean percentage difference in outcome per 1 standard deviation increase in IGF. Findings did not differ when IGF measures were modeled as either *z*‐scores or as absolute values. Second, associations between change in IGF‐I and II and fat and lean mass were examined using conditional change models—calculated change in IGF was included in models that also included the baseline IGF concentration at 53 years. Change scores were converted to sex‐specific *z*‐scores to aid interpretation of model coefficients. Given previous evidence for sex differences in associations 26, sex interaction terms were tested—where significant interactions were found (*P* < 0.05), models were conducted separately in each sex; otherwise models were adjusted for sex.

Associations with lean mass could be confounded by fat mass, as changes in fat mass typically lead to adaptive changes in lean mass 27. As such, models using lean mass as an outcome were additionally adjusted for fat mass. The change models were also additionally adjusted for further potential confounders at age 53 identified a‐priori: household occupational class, smoking status (categorized as non‐smoker or light 1, 2, 3, 4, 5, 6, 7, 8, 9, 10, moderate 11, 12, 13, 14, 15, 16, 17, 18, 19, 20, or heavy smoker (>20 cigarettes per day)) and menopausal status (categorized as pre‐, peri‐, or post‐menopausal, hysterectomy with or without hormone replacement therapy, other hormone replacement therapy user, or other reason for menstrual period cessation) 28. Complete case analyses were performed to examine IGF and body composition associations: 594 men and 644 women had complete body composition and IGF data at both ages; of these, 588 men and 623 women also had complete data for all potential confounders.

### Additional and sensitivity analyses

To examine the possibility of reverse or bi‐directionality, change models with fat mass were repeated with additional adjustment for BMI at 53 (as DXA measures were not available at 53), and analyses were conducted in which BMI (or change in BMI) was the exposure, and IGF‐I or II the outcome. As IGF‐I and physical activity may influence muscle mass synergistically 7, we also examined whether physical activity modified the association between IGF‐I and lean mass by including an interaction term with leisure time physical activity at both 53 and 60‐64 years (categorized as no participation, 1‐4 episodes, and ≥5 episodes in the previous 4 weeks). To examine the extent to which associations between IGF and body composition were driven by height, we also conducted analyses without adjustment for height. To examine whether associations between IGFs and body composition outcomes were non‐linear, we examined plots of the data and compared linear models with models additionally including quadratic terms for IGFs. We also examined whether associations between changes in IGFs and body composition were non‐linear, by repeating analyses using change score variables converted into quartiles. These categorical models were then compared with linear models using likelihood ratio tests.

## Results

### Descriptive analyses

At both 53 and 60‐64 years, men had higher IGF‐I but lower IGF‐II concentrations than women (Table 1). In both sexes, mean IGF‐I and II concentrations were lower at 60‐64 than 53 years—by 12.8% for IGF‐I and 12.5% for IGF‐II; larger declines were seen in IGFBP3 and therefore the mean IGF‐I:IGFBP3 ratio was higher at 60‐64 years. Most, but not all, participants showed a decline in IGF‐I (66%) and IGF‐II (65%) concentrations (Supporting Information Figure 1). Women had greater whole body fat and less lean mass than men, and a lower android:gynoid ratio.

**Table 1:** Summary of body composition and IGF concentrations by sex

	Men, mean (SD or IQR)	Women, mean (SD or IQR)	*P* a
***Body composition at 60‐64 y***	*N* = 746	*N* = 812	
**Fat mass index (kg m^−2^)**	12.02 (3.63)	16.20 (4.97)	<0.001
**Android fat mass (kg)**	2.47 (0.96)	2.34 (0.98)	0.02
**Gynoid fat mass (kg)**	3.73 (1.00)	5.11 (1.41)	<0.001
**Android:gynoid ratio**	65.16 (15.50)	44.93 (12.04)	<0.001
**Appendicular lean mass index (kg m^−2^)**	8.02 (0.95)	6.19 (0.87)	<0.001
***IGF concentration, age***	*N* = 744	*N* = 798	
**IGF‐I (ng ml^−1^), 53 y**	211.2 (66.3)	194.6 (67.9)	<0.001
**IGF‐I (ng ml^−1^), 60‐64y**	185.7 (59.9)	168.2 (58.6)	<0.001
**Δ IGF‐I (ng ml^−1^)**	−25.5 (−60, 14)	−26.4 (−67, −24)	0.80
**IGF‐II (ng ml^−1^), 53 y**	747.7 (254.2)	796.6 (247.6)	<0.001
**IGF‐II (ng ml^−1^), 60‐64 y**	647.0 (308.0)	703.0 (291.5)	<0.001
**Δ IGF‐II (ng ml^−1^)**	−100.6 (−321, 99)	−93.5 (−358, 115)	0.70
**IGFBP‐3 (ng ml^−1^), 53 y**	4781.7 (1083.2)	4835.9 (1122.5)	0.30
**IGFBP‐3 (ng ml^−1^), 60‐64 y**	3219.5 (832.4)	3457.7 (831.5)	<0.001
**Δ IGFBP‐3 (ng ml^−1^)**	−1562.2 (−2244, −881)	−1378.1 (−2142, −592)	<0.001
**IGF‐I:IGFBP‐3, 53 y**	4.6 (1.7)	4.2 (1.9)	<0.001
**IGF‐I:IGFBP‐3, 60‐64 y**	6.0 (2.5)	5.0 (1.5)	<0.001
**Δ IGF‐I:IGFBP‐3**	1.4 (0.17, 2.6)	0.8 (−0.35, 1.9)	<0.001

a Comparison between sexes using *t* tests; IGF‐I/IGFBP‐3 ratio was multiplied by 100; analyses restricted to participants with valid data for all body composition outcomes or all hormone measures at both ages.

IGF‐I showed moderately positive correlations with IGF‐II and IGFBP‐3 at both 53 and 60‐64 years (Supporting Information Table 1).

### Associations between IGFs and fat mass

IGF‐I at 53 (in women) and 60‐64 years (both sexes) was inversely associated with fat mass at 60‐64 (Table 2). Conversely, IGF‐II at 53 years was positively associated with fat mass, while IGF‐II at 60‐64 years was inversely associated with fat mass. Associations between IGF‐I or II with BMI and android:gynoid ratio at 60‐64 years was generally similar to those with fat mass index (Supporting Information Table 2).

**Table 2:** Mean percentage differences in fat mass and android:gynoid ratio (95% CI) at age 60‐64 years per 1 standard deviation increase in IGF‐I and IGF‐II at 53 and 60‐64 years

			Fat mass index			Android:gynoid fat mass ratio		
		*N*	β (95% CI)	*P*	*P* (sex interaction)	β (95% CI)	*P*	*P* (sex interaction)
**IGF‐I at 53y**	Men	627	0.38 (−2.04, 2.79)	0.76	<0.01	−0.30 (−1.74, 1.13)	0.68	0.16
	Women	704	−4.06 (−6.26, −1.85)	<0.001				
**IGF‐I at 60‐64 y**		1,434	−2.12 (−3.71, −0.52)	<0.01	0.94	−0.73 (−2.11, 0.66)	0.30	0.15
**Δ IGF‐I**		1,211	−1.86 (−4.02, 0.30)	0.09	0.61	−1.33 (−3.23, 0.57)	0.17	0.24
Δ IGF‐I, adjusted*		1,211	−1.56 (−3.71, 0.59)	0.16	0.51	−0.96 (−2.86, 0.94)	0.32	0.19
**IGF‐II at 53y**		1,331	3.24 (1.61, 4.86)	<0.001	0.26	2.71 (1.28, 4.14)	<0.001	0.30
**IGF‐II at 60‐64 y**		1,434	−1.55 (−3.15, 0.05)	0.06	0.09	0.76 (−0.63, 2.14)	0.28	0.55
**Δ IGF‐II**		1,211	−2.01 (−4.05, 0.03)	0.05	0.10	0.27 (−1.53, 2.06)	0.77	0.26
**Δ IGF‐II, adjusted** a		1,211	−1.94 (−3.96, 0.08)	0.06	0.11	0.26 (−1.53, 2.04)	0.78	0.27

Note: Sex‐specific findings shown where *P* (sex interaction)<0.05; otherwise, models are adjusted for sex. Δ change between 53 and 60‐64 years—analyses adjusted for hormone concentration at 53 years.

a Adjusted for highest household occupational class, smoking, and menopausal status at age 53 years.

Greater decline in IGF‐I between 53 to 60‐64 years was weakly and non‐significantly associated with higher fat mass, and higher android:gynoid ratio at 60‐64 years (Table 2). Greater decline in IGF‐II was associated with higher fat mass, but not android: gynoid ratio. These associations were similar albeit partly attenuated when additional adjustment was made for potential confounders (Table 2). Associations of IGFBP‐3 and IGF‐I: IGFBP‐3 with these outcomes are shown in Supporting Information Table 3.

### Associations between IGFs and lean mass

After adjustment for fat mass, IGF‐I at 53 was positively associated with appendicular lean mass at 60‐64 years, while associations between IGF‐I at 60‐64 years and lean mass were weak and not significant (Table 3). After adjustment for fat mass, IGF‐II was not associated with lean mass in either sex, and nor were changes in IGF‐I or II.

**Table 3:** Mean percentage differences in appendicular lean mass (95% CI) at age 60‐64 years per 1 standard deviation increase in IGF‐I and IGF‐II at 53 and 60‐64 years

			Appendicular lean mass index, unadjusted			Appendicular lean mass index, adjusted for fat mass index		
		*N*	*β* (95% CI)	*P*	*P* (sex interaction)	*β* (95% CI)	*P*	*P* (sex interaction)
**IGF‐I at 53 y**	Men	627	0.97 (0.05, 1.89)	0.04	<0.01	0.65 (0.10, 1.21)	0.02	0.19
	Women	704	−1.02 (−2.03, −0.01)	0.05				
**IGF‐I at 60‐64y**		1,434	−0.39 (−1.05, 0.28)	0.25	0.86	0.26 (−0.28, 0.80)	0.34	0.87
**Δ IGF‐I**		1,211	−0.75 (−1.66, 0.16)	0.11	0.62	−0.25 (−0.98, 0.49)	0.51	0.65
**Δ IGF‐I, adjusted** a		1,211	−0.69 (−1.60, 0.22)	0.14	0.70	−0.28 (−1.01, 0.46)	0.46	0.83
**IGF‐II at 53 y**		1,331	0.95 (0.26, 1.63)	<0.01	0.33	0.23 (−0.33, 0.79)	0.41	0.69
**IGF‐II at 60‐64 y**		1,434	−0.34 (−1.01, 0.32)	0.31	0.84	0.10 (−0.44, 0.64)	0.72	0.33
**Δ IGF‐II**		1,211	−0.43 (−1.29, 0.43)	0.33	0.56	0.11 (−0.58, 0.81)	0.75	0.53
**Δ IGF‐II, adjusted** a		1,211	−0.33 (−1.19, 0.53)	0.45	0.67	0.19 (−0.50, 0.89)	0.59	0.42

Note: Sex‐specific findings shown where *P* (sex interaction) < 0.05; otherwise, models are adjusted for sex. Δ change between 53 and 60‐64 years—analyses adjusted for hormone concentration at 53 years.

a Adjusted for highest household occupational class, smoking, and menopausal status at age 53 years.

### Additional and sensitivity analyses

Associations between declines in IGF‐I and II and fat mass were similar after additional adjustment for BMI at 53 years (*P* = 0.04 for IGF‐I and *P* = 0.13 for IGF‐II; Supporting Information Table 4). Greater gain in BMI from 53 to 60‐64 years (modeled as the exposure) was weakly associated with lower IGF‐I at 60‐64 years (modeled as the outcome), but not with IGF‐II at 60‐64 years (Supporting Information Table 5).

There was no evidence for effect modification by physical activity in the association between IGF‐I and lean mass (*P* values for interaction term>0.4 at both 53 and 60‐64 years). For all outcomes, conclusions did not differ when not adjusting for adult height. There was no substantive evidence for deviation from linearity with IGF at each age, nor with change in IGF‐I or II (*P* values from likelihood ratio tests >0.35 in all cases).

## Discussion

Longitudinal data from a large British birth cohort study showed evidence for declines in both IGF‐I and II concentrations during late midlife (53 to 60‐64 years), which were associated with higher fat mass at 60‐64 years. While IGF‐I at 53 years was positively associated with lean mass at 60‐64 years, there was little evidence that changes in IGF‐I and II, per se, were associated with lean mass in either sex.

Our findings add substantially to previous cross‐sectional evidence for lower IGF‐I concentrations in older versus younger adults 15, 16, 17, 18, and one small longitudinal study which reported a decline in IGF‐I during mid‐late life among participants enrolled in a colonoscopy study (*N* = 143) 29. The results also add to previous cross‐sectional studies conducted on smaller samples which reported equivocal findings on the relationship between IGF‐I and body composition. For example, in a cross‐sectional study of Dutch older adults, IGF‐I was inversely associated with BMI, and weakly inversely associated with fat and lean mass in men, but not women 26. In other cross‐sectional studies of adults, IGF‐I was inversely 30, positively 31, or not associated with lean mass 32, 33; and inversely 32, or not associated with fat mass 30, 31, 33. Previous studies have also found evidence that the IGF‐I and BMI cross‐sectional association is non‐linear, with those with highest or lowest BMI having lower IGF‐I 21, 22. However, there was little evidence for this in our models using either fat mass or BMI as the outcome (Supporting Information Figures 2 and 3) with mean IGF‐I levels declining with increasing quintiles of body mass or fat mass index.

Taken together, these results provide some evidence to support the roles of IGF‐I and II in the regulation of fat mass. The weak magnitude of associations (and non‐significant *P* values found in some cases) may be explained by the contribution of other influences on fat mass which we were unable to control for, and by imprecision in the extent to which a single measure of circulating IGF reflects long‐term concentrations (e.g., due to day‐do‐day variation) and bioactivity. The presence of feedback loops may also weaken observed associations. For example, while increases in IGF‐I may reduce fat mass levels, higher IGF‐I would also down‐regulate growth hormone secretion, which in turn would result in lower IGF‐I. Associations between IGF‐I and fat mass could also be explained in part by reverse causation or bi‐directionality, as obese subjects are known to have relative growth hormone insensitivity compared with nonobese subjects 8. Although associations between change in IGF‐I and fat mass were not substantively attenuated after adjustment for BMI at 53 year, greater gains in BMI were (weakly) associated with lower IGF‐I. Finally, IGF‐I could simply be a surrogate marker for the direct actions of growth hormone on mature adipocytes 8.

Lack of association, or weak association, between changes in total serum IGF concentrations and lean mass could suggest that IGF‐I or II are not important regulators of adult lean mass. This is contrary to reported findings from some studies which reported higher adult protein intake, a likely determinant of muscle mass, associated with higher IGF‐I concentrations 34. However, our findings do not preclude the importance of IGF‐I and II in regulating muscle growth in early life, nor local IGF actions on adult lean mass—some experimental studies have shown that exercise increases local (muscle) IGF‐I but not total serum IGF‐I concentrations 35.

Unlike total IGF‐I concentrations, mean IGF‐I:IGFBP‐3 ratio increased between 53 to 60‐64 years due to the relatively larger decline in IGFBP‐3. IGFBP‐3 is one of six binding proteins that carry IGF‐I in circulation. If IGFBP‐3 acts as an inert carrier protein, then the IGF‐I:IGFBP‐3 ratio could better indicate biologically available IGF‐I than total IGF‐I concentration. However, evidence for direct biological actions of IGFBP‐3 suggests otherwise—IGFBP‐3 and other IGF binding proteins have their own cellular actions which include enhancing IGF activity 36. As such, associations between IGF‐I:IGFBP‐3 ratio and outcomes should be interpreted with caution. However, directly measured “free” and/or bioactive IGF‐I or II may decline with age, and its decline may relate more closely to body composition outcomes than total measures. This warrants investigation in future studies although at present there are no universally accepted standardized methods for measuring “free” or bioactive concentrations of IGFs and the interpretation of these measurements is still subject to discussion.

It is unclear what up‐stream factors cause the observed age‐related declines in IGF‐I and II. IGF‐I has been hypothesized to be a biomarker which mediates the roles of physical activity (and other factors) on body composition and health outcomes 37. However, it remains unclear if this is the case. While studies have consistently found greater milk consumption in early life is associated with lower IGF‐I concentration in adulthood 38, studies examining the adulthood determinants of IGF have yielded equivocal findings [e.g., in relation to reported energy intake and physical activity level 20]. These determinants are being investigated in the NSHD and warrant investigation in other cohorts.

Strengths of this study include the accurate measures of fat and lean mass in both sexes, and repeat measures of both IGF‐I and II. The single DXA measures obtained at 60‐64 years however precluded the analysis of how changes in IGF relate to change in fat and lean mass. Intra‐assay coefficients of variation were low, which limited measurement error; while inter‐assay coefficient variations were larger, the ranking of participant IGF values was unlikely to be substantially affected as assay results were calibrated using control samples.

Limitations of study include loss to follow‐up, which despite the large sample size reduced statistical power and may have introduced bias (if the exposure‐outcome association differed in the sample lost to follow‐up). Methodological differences in blood sample collection at 53 and 60‐64 years could have affected the results obtained—the longer storage time of samples collected at 53 years could have resulted in IGF degradation, which would attenuate the declines in IGF‐I and II observed with age. Conversely, the liquid citrate used at 60‐64 years may have reduced IGF concentrations. Blood samples at 53 years were obtained during home visits, which likely resulted in a longer time to freezing compared with the 60‐64 year samples. However, a methodological study suggested that time to freezing did not substantially affect IGF‐I concentrations 39, and IGF‐I and II concentrations at 53 years did not differ by time of day at sampling (morning, afternoon, or evening; *P* > 0.3 in all cases). More than two time points of measurement would provide more informative data on the trajectories of change in IGF concentrations with age.

Findings from the present study add substantially to our understanding of the age‐related changes in IGF‐I and II, and their consequences for body composition. However, the implications of these findings are not straightforward. Lowering fat mass levels by pharmacological supplementation of IGF (or growth hormone) is likely to be unwarranted given expected increases in cancer risk 40. Rather, behavioral or early life interventions acting on the up‐stream determinants of IGF‐I and II may potentially lessen their decline with age in later mid‐life, and in turn could limit the accumulation of fat mass during this period of ageing.

## Conclusion

Using longitudinal data from a British birth cohort study, evidence was found for declines in IGF‐I and II concentrations from 53 to 60‐64 years, and greater declines were weakly associated with higher fat mass at 60‐64 years. There was little evidence that changes in IGF‐I and II were associated with lean mass.

## Acknowledgments

The authors thank Dr. Andrew Wong and other members of the MRC National Survey for Health and Development scientific and data collection team at the following centers in the United Kingdom: MRC Unit for Lifelong Health and Ageing; Wellcome Trust Clinical Research Facility 505 (CRF) Manchester and the Department of Clinical Radiology at the Central Manchester University Hospitals National Health Service Foundation Trust; Wellcome Trust CRF and Medical Physics at the Western General Hospital in Edinburgh; Wellcome Trust CRF and the Department of Nuclear Medicine at University Hospital Birmingham; Wellcome Trust CRF and the Department of Nuclear Medicine at University College London Hospital; CRF and the Department of Medical Physics at the University Hospital of Wales; and CRF and Twin Research Unit at St. Thomas' Hospital London. They also thank members of the MRC National Survey for Health and Development Bone and Muscle Project Management Group and those involved in completing IGF assays (including Dr. Caroline Jarrett).

## Supporting information

Supporting Information
