# Supplementary material for: Changes in insulin‐like growth factor‐I and ‐II associated with fat but not lean mass in early old age
Source: Obesity (Silver Spring). 2015 Feb 3;23(3):692–8. doi: 10.1002/oby.21002 (PMC4737231; doi:10.1002/oby.21002)
Supplement: Supplementary file 1 — Supporting Information [file OBY-23-692-s001.docx]

Supplementary Table 1. Correlations between IGF-I, IGF-II, and IGFBP-3 at 53 and 60-64y

a) Men (n=744)

| IGF-I, 60-64y | 0.47 |  |  |  |  |  |  |
| --- | --- | --- | --- | --- | --- | --- | --- |
| IGF-II, 53y | 0.27 | 0.17 |  |  |  |  |  |
| IGF-II, 60-64y | 0.11 | 0.24 | 0.20 |  |  |  |  |
| IGFBP-3, 53y | 0.30 | 0.25 | 0.37 | 0.19 |  |  |  |
| IGFBP-3, 60-64y | 0.32 | 0.43 | 0.24 | 0.24 | 0.37 |  |  |
| IGF-I: BP3, 53y | 0.66 | 0.24 | -0.01 | 0.00 | -0.40 | 0.03 |  |
| IGF-I: BP3, 60-64y | 0.14 | 0.42 | -0.02 | 0.00 | -0.05 | -0.38 | 0.16 |
|  | IGF-I,  53y | IGF-I,  60-64y | IGF-II,  53y | IGF-II,  60-64y | IGFBP-3,  53y | IGFBP-3,  60-64y | IGF-I: BP3,  53y |

a) Women (n=798)

| IGF-I, 60-64y | 0.40 |  |  |  |  |  |  |
| --- | --- | --- | --- | --- | --- | --- | --- |
| IGF-II, 53y | 0.19 | 0.11 |  |  |  |  |  |
| IGF-II, 60-64y | 0.07 | 0.24 | 0.13 |  |  |  |  |
| IGFBP-3, 53y | 0.31 | 0.31 | 0.29 | 0.22 |  |  |  |
| IGFBP-3, 60-64y | 0.29 | 0.51 | 0.23 | 0.28 | 0.32 |  |  |
| IGF-I: BP3, 53y | 0.60 | 0.14 | 0.01 | -0.09 | -0.42 | 0.06 |  |
| IGF-I: BP3, 60-64y | 0.20 | 0.67 | -0.05 | 0.04 | 0.08 | -0.25 | 0.11 |
|  | IGF-I,  53y | IGF-I,  60-64y | IGF-II,  53y | IGF-II,  60-64y | IGFBP-3,  53y | IGFBP-3,  60-64y | IGF-I: BP3,  53y |

Supplementary Table 2. Mean percentage differences in body mass index (kg/m^2^) (95% CI) at age 60–64 years per 1 standard deviation increase in IGF-I and IGF-II at 53 and 60-64 years

|  |  |  |  | | | |  |  |
| --- | --- | --- | --- | --- | --- | --- | --- | --- |
|  |  | N | β (95% CI) | P | P sex interaction |  |  |  |
| IGF-I (ng/ml) at 53y | Men | 627 | 0.62(-0.48, 1.72) | 0.27 | 0.01 |  |  |  |
|  | Women | 704 | -2.09(-3.36, -0.83) | <0.01 |  |  |  |  |
| IGF-I (ng/ml) at 60-64y |  | 1434 | -0.90(-1.72, -0.08) | 0.03 | 0.92 |  |  |  |
| ∆ IGF-I (ng/ml) |  | 1211 | -0.93(-2.05, 0.20) | 0.11 | 0.72 |  |  |  |
|  |  |  |  |  |  |  |  |  |
| IGF-II (ng/ml) at 53y |  | 1331 | 1.73(0.89, 2.57) | <0.001 | 0.34 |  |  |  |
| IGF-II (ng/ml) at 60-64y |  | 1434 | -0.53(-1.35, 0.30) | 0.21 | 0.18 |  |  |  |
| ∆ IGF-II (ng/ml) |  | 1211 | -0.66(-1.72, 0.40) | 0.22 | 0.10 |  |  |  |

Note: Sex-specific findings shown where P(sex interaction)<0.05, otherwise models are adjusted for sex; ; ∆ change between 53 and 60-64 years—analyses adjusted for hormone concentration at 53 years.

Supplementary Table 3. Mean percentage differences in body composition outcomes (95% CI) at age 60–64 years per 1 standard deviation increase in conditional change in IGFBP-3 and IGF-I: IGFBP-3 from 53 to 60-64 years

|  |  |  | Fat mass index |  |  | Android: gynoid fat mass ratio |  |  |
| --- | --- | --- | --- | --- | --- | --- | --- | --- |
|  |  | N | β (95% CI) | P | P sex interaction | β (95% CI) | P | P sex interaction |
| IGFBP-3 at 53y | Men | 627 | 3.31(0.91, 5.71) | <0.01 | <0.01 | 1.90(0.46, 3.34) | <0.01 | 0.38 |
|  | Women | 704 | -1.68(-3.91, 0.55) | 0.14 |  |  |  |  |
| IGFBP-3 at 60-64y |  | 1434 | -1.05(-2.65, 0.55) | 0.20 | 0.92 | 1.06(-0.32, 2.44) | 0.13 | 0.91 |
| ∆ IGFBP-3 |  | 1211 | -1.62(-4.14, 0.91) | 0.21 | 0.53 | 0.73(-1.48, 2.94) | 0.52 | 0.69 |
|  |  |  |  |  |  |  |  |  |
| IGF-I: IGFBP-3 at 53y |  | 1331 | -1.95(-3.59, -0.31) | 0.02 | 0.78 | -1.17(-2.61, 0.27) | 0.11 | 0.68 |
| IGF-I: IGFBP-3 at 60-64y |  | 1434 | -1.20(-2.80, 0.41) | 0.14 | 0.78 | -1.07(-2.45, 0.31) | 0.13 | 0.49 |
| ∆ IGF-I: IGFBP-3 |  | 1211 | -1.30(-3.47, 0.87) | 0.24 | 0..60 | -1.47(-3.38, 0.43) | 0.13 | 0.86 |

|  |  |  | Appendicular lean mass index, unadjusted | | | Appendicular lean mass index, adjusted for fat mass index | | |
| --- | --- | --- | --- | --- | --- | --- | --- | --- |
|  |  | N | β (95% CI) | P | P sex interaction | β (95% CI) | P | P sex interaction |
| IGFBP-3 at 53y | Men | 627 | 1.39(0.47, 2.31) | <0.01 | 0.001 | 0.83(0.02, 1.63) | 0.05 | 0.05 |
|  | Women | 704 | -0.89(-1.90, 0.12) | 0.08 |  | -0.34(-1.11, 0.44) | 0.40 |  |
| IGFBP-3 at 60-64y |  | 1434 | 0.00(-0.67, 0.66) | 0.99 | 0.44 | 0.40(-0.14, 0.94) | 0.14 | 0.37 |
| ∆ IGFBP-3 |  | 1211 | -0.42(-1.49, 0.64) | 0.43 | 0.71 | 0.16(-0.70, 1.01) | 0.72 | 0.52 |
|  |  |  |  |  |  |  |  |  |
| IGF-I: IGFBP-3 at 53y |  | 1331 | -0.08(-0.77, 0.62) | 0.83 | 0.99 | 0.50(-0.06, 1.06) | 0.08 | 0.84 |
| IGF-I: IGFBP-3 at 60-64y |  | 1434 | -0.35(-1.02, 0.31) | 0.30 | 094 | -0.01(-0.55, 0.53) | 0.98 | 0.91 |
| ∆ IGF-I: IGFBP-3 |  | 1211 | -0.31(-1.22, 0.61) | 0.51 | 0.92 | 0.03(-0.71, 0.76) | 0.95 | 0.59 |

Note: Sex-specific findings shown where P(sex interaction)<0.05, otherwise models are adjusted for sex; ∆ change between 53 and 60-64 years—analyses adjusted for hormone concentration at 53 years.

Supplementary Table 4. Mean percentage differences in fat mass (95% CI) at 60–64 years per 1 standard deviation increase in change in IGF-II and IGF-II between 53 and 60-64 years, adjusted for hormone concentration and body mass index (kg/m^2^) at 53 years

|  |  |  |  |  |  |  |
| --- | --- | --- | --- | --- | --- | --- |
|  | | |  | Fat mass index | | |
|  | | | N | β (95% CI) | P | P sex interaction |
| ∆ IGF-I | | | 1211 | -1.51(-2.91, -0.10) | 0.04 | 0.50 |
| ∆ IGF-II | | | 1211 | -1.03(-2.37, 0.31) | 0.13 | 0.15 |

Note: #P-value for sex interaction term (#before and ##after adjustment for fat mass index).

Supplementary Table 5. Mean percentage differences in IGF-I and II at 60-64 years (95% CI) per 1 standard deviation increase in change in BMI (kg/m^2^) between 53 and 60-64 years

|  |  |  | |  | |  |  | |  | | |  |  |
| --- | --- | --- | --- | --- | --- | --- | --- | --- | --- | --- | --- | --- | --- |
|  |  | IGF-I at 60-64y |  | |  | | IGF-II at 60-64y |  | |  |  |  |  |
|  | N | β (95% CI) | P | | P sex  interaction | | β (95% CI) | P | | P sex  interaction |  |  |  |
| BMI at 53y | 1331 | -2.57(-4.39, -0.74) | <0.01 | | 0.70 | | -0.86(-3.26, 1.54) | 0.48 | | 0.82 |  |  |  |
| BMI at 60-64y | 1434 | -2.88(-4.66, -1.11) | <0.01 | | 0.79 | | -1.57(-3.89, 0.76) | 0.19 | | 0.33 |  |  |  |
| ∆ BMI | 1211 | -1.52(-3.35, 0.32) | 0.11 | | 0.72 | | -0.30(-2.71, 2.11) | 0.81 | | 0.09 |  |  |  |

Supplementary Figure 1. Histograms of absolute difference scores between IGF concentrations at 53 and 60-64 years

a) Men

b) Women

Supplementary figure 2. Scatter plots (with linear lines of best fit) of IGF-I at 60-64 years with adiposity measures at 60-64 years

Fat mass index:

Body mass index:

Supplementary figure 3. Mean IGF-I concentrations (95% confidence intervals) stratified by body mass index (BMI) and fat mass index quintiles (all measured at 60-64 years)


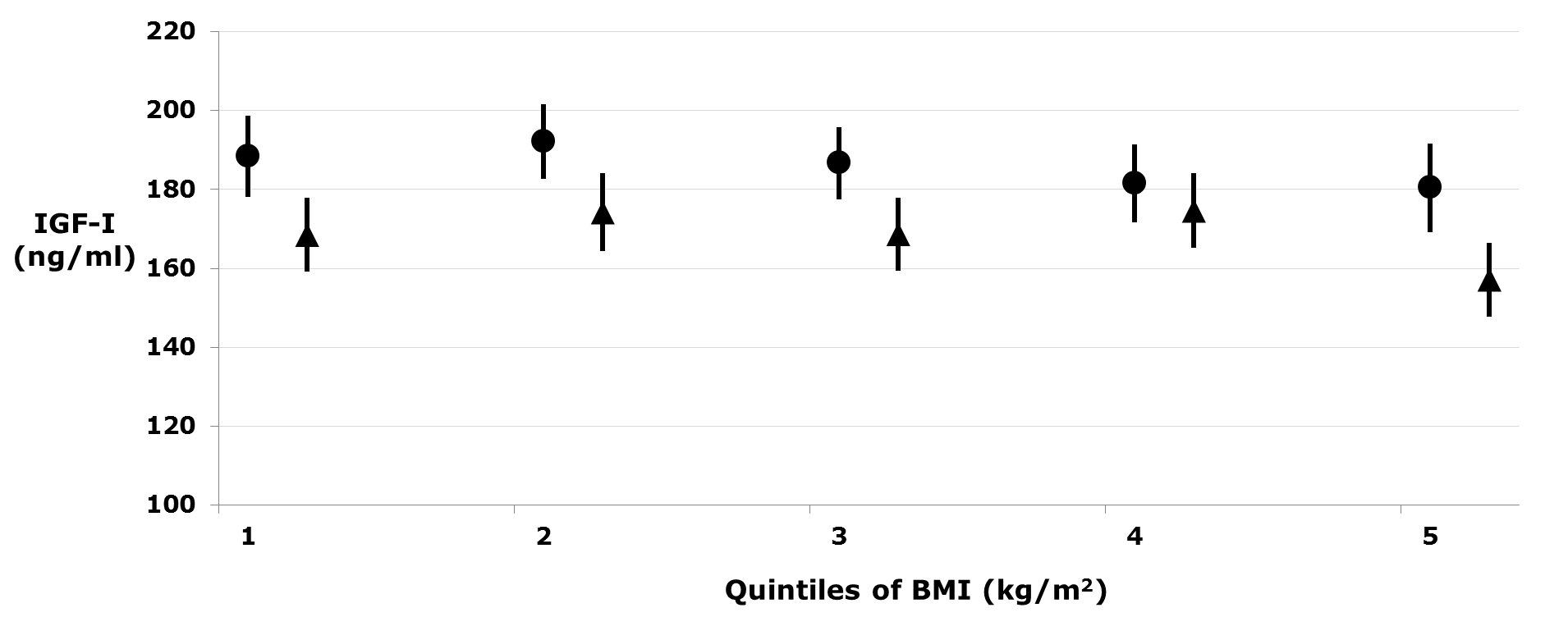


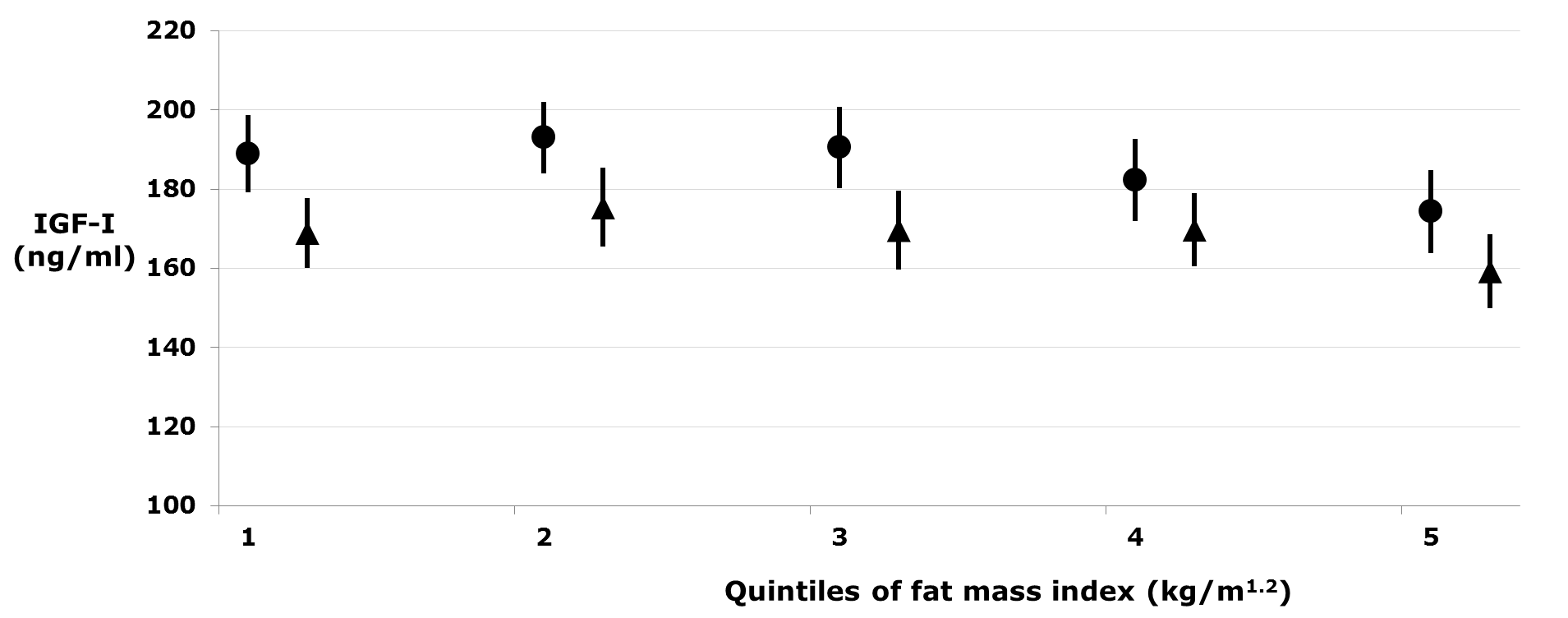


Note: men shown in circles, women in triangles. The range of values within each quintile is:

BMI:

Men: <24.30, 24.30–26.46, 26.47–28.27, 28.28-30.91, >30.91;

Women: <23.40, 23.40–25.46, 25.47-27.57, 27.58-31.40, >31.40

Fat mass index:

Men: <9.01, 9.01-10.91, 10.92-12.68, 12.69-14.90, >14.90

Women: <11.94, 11.94-14.01, 14.02-16.55, 16.56-20.17, >20.17
